# Role of the β_2_-adrenergic receptor in podocyte injury and recovery

**DOI:** 10.1007/s43440-024-00594-5

**Published:** 2024-04-26

**Authors:** Ehtesham Arif, Ashish K. Solanki, Bushra Rahman, Bethany Wolf, Rick G. Schnellmann, Deepak Nihalani, Joshua H. Lipschutz

**Affiliations:** 1 Present Address: Department of Medicine, Nephrology Division, Medical University of South Carolina, Clinical Science Building 822N, 96 Jonathan Lucas Street, Charleston, SC 29425 USA; 2 Department of Public Health Sciences, Medical University of South Carolina, Charleston, SC USA; 3 Department of Pharmacology and Toxicology, University of Arizona, Tucson, AZ USA; 4 Southern Arizona VA Health Care System, Tucson, AZ USA

**Keywords:** Acute kidney injury, β_2_-adrenergic receptor, Formoterol

## Abstract

**Background:**

Podocytes have a remarkable ability to recover from injury; however, little is known about the recovery mechanisms involved in this process. We recently showed that formoterol, a long-acting β_2_-adrenergic receptor (β_2_-AR) agonist, induced mitochondrial biogenesis (MB) in podocytes and led to renoprotection in mice. However, it is not clear whether this effect was mediated by formoterol acting through the β_2_-AR or if it occurred through “off-target” effects.

**Methods:**

We genetically deleted the β_2_-AR specifically in murine podocytes and used these mice to determine whether formoterol acting through the podocyte β_2_-AR alone is sufficient for recovery of renal filtration function following injury. The podocyte-specific β_2_-AR knockout mice (β_2_-AR^fl/fl^/PodCre) were generated by crossing β_2_-AR floxed mice with podocin Cre (B6.Cg-Tg(NPHS2-cre)295Lbh/J) mice. These mice were then subjected to both acute and chronic glomerular injury using nephrotoxic serum (NTS) and adriamycin (ADR), respectively. The extent of injury was evaluated by measuring albuminuria and histological and immunostaining analysis of the murine kidney sections.

**Results:**

A similar level of injury was observed in β_2_-AR knockout and control mice; however, the β_2_-AR^fl/fl^/PodCre mice failed to recover in response to formoterol. Functional evaluation of the β_2_-AR^fl/fl^/PodCre mice following injury plus formoterol showed similar albuminuria and glomerular injury to control mice that were not treated with formoterol.

**Conclusions:**

These results indicate that the podocyte β_2_-AR is a critical component of the recovery mechanism and may serve as a novel therapeutic target for treating podocytopathies.

**Supplementary Information:**

The online version contains supplementary material available at 10.1007/s43440-024-00594-5.

## Introduction

Podocytes are terminally differentiated cells and along with fenestrated endothelial cells and glomerular basement membrane (GBM) constitute the glomerular filtration barrier. This barrier provides selective passage for macromolecules into the urinary space [1, 2]. Podocyte damage is a common occurrence in many glomerular diseases such as focal and segmental glomerulosclerosis (FSGS) which can manifest as nephrotic syndrome [2, 3]. Therefore, significant effort is being spent on identifying strategies to preserve or recover podocyte function. Accordingly, studies have shown that following injury glomerular filtration function can be preserved or restored through drug-induced recovery of podocytes [4–6]. Unfortunately, the podocyte-targeted therapies that have been developed have shown limited therapeutic potential [4, 6]. Recent studies, including ours, have shown that use of β_2_-AR agonists may offer therapeutic benefit in restoring podocyte function [7–9].

The β_2_-AR, which is widely expressed and a member of the G-protein-coupled receptor (GPCR) family, can be activated in an agonist-induced fashion [8, 10]. Genetic polymorphisms of the β_2_-AR in humans are associated with a differential response to β_2_-agonists [11, 12]. Studies involving desensitization and re-sensitization of this receptor have shown that various β_2_-AR agonists may offer clinical benefits in treating asthma, cardiovascular, and other diseases [13, 14]; however, little is known about their role in glomerular diseases affecting podocyte function. Since we first demonstrated that β_2_-AR agonists in mice enhanced podocyte recovery following injury and also showed that the β_2_-AR was expressed in podocytes [15], we wanted to further investigate whether β_2_-AR agonists act though the β_2_-AR in podocytes or in an “off-target” manner. We therefore generated podocyte-specific β_2_-AR knockout mice and used them to determine the pathophysiological significance of the β_2_-AR in podocyte function.

## Material and methods

### RNA-seq data

RNA-seq data previously described [15, 16] (GEO# GSE124622 & GSE117669) was processed for determining the expression of β_2_-AR in various injury models. The differential expression of β_2_-AR was based on p values of < 0.05, when adjusted using the Benjamini and Hochberg’s approach.

### Generation of β_2_-AR knockout mice

**Fig. 1:**
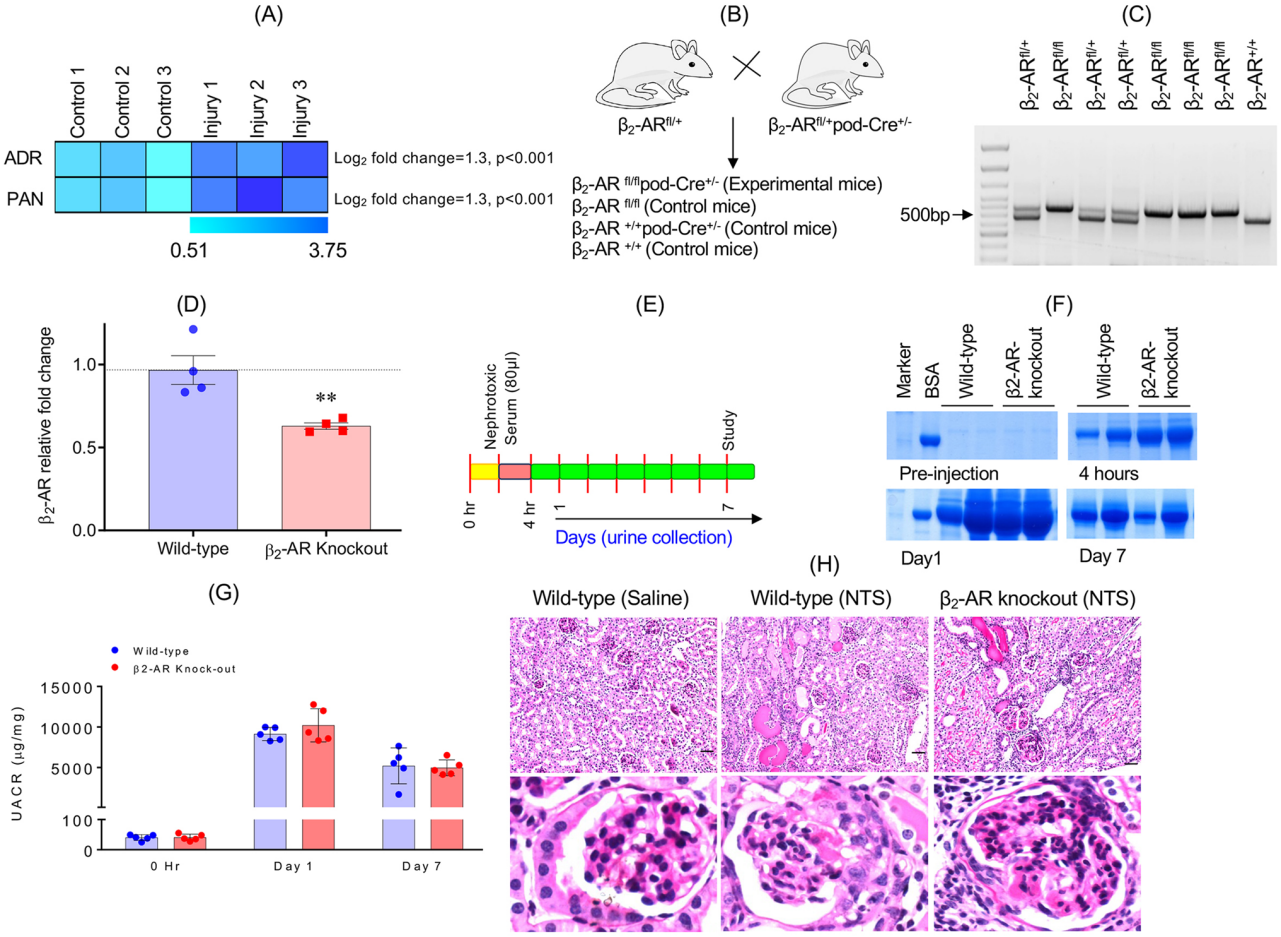
Generation of podocyte-specific beta 2 adrenergic receptor (β2-AR) knockout mice. **A** The mRNA profile of podocytes treated with adriamycin (ADR) and puromycin aminonucleoside (PAN) showed increased expression of the β_2_-AR gene following injury. Log_2_ fold changes in the β_2_-AR gene expression were significant (adjusted p values < 0.05). **B** Schematic for the generation of the control (wild-type) and experimental mice is shown. **C** β_2_-AR knockout mice were genotyped using gene specific primers. Control mice displayed a band of ~ 500 bp while the β_2_-AR knockout mice displayed a band of ~ 550 bp. Both these bands were present in the heterozygous mice. **D** qPCR analysis showed significant down regulation of the β_2_-AR in glomeruli isolated from β_2_-AR knockout mice. Data are presented as mean ± standard error of mean (SEM). **P ≤ 0.0001, wild-type vs β_2_-AR knockout mice, Student’s t test (2 tailed; t_3.3_ =  − 3.807). n = 4 mice per group. **E** Schematic of the experimental plan. **F** Sodium dodecyl-sulfate polyacrylamide gel electrophoresis (SDS-PAGE) analysis of urine samples showed no significant difference in albuminuria pre- and post-nephrotoxic serum (NTS) injury between wild-type and β_2_-AR knockout mice. **G** The urine albumin/creatinine ratio (UACR) also showed no significant difference in albuminuria between wild-type and β_2_-AR knockout mice. Data are presented in mean ± SEM and analyzed using a two-way analysis of variance (ANOVA) (main effect time: F_2,16_ = 123.9, p < 0.0001); main effect β_2_-AR knockout: F_1,8_ = 0.8453, p = 0.5716; interaction: F_2,16_ = 0.6400, p = 0.5403) with a Holm-Sidak adjustment for p values. *P* > 0.05, wild-type vs. β_2_-AR knockout mice. n = 5 mice per group. **H** Representative histological sections of the kidneys by hematoxylin and eosin (H&E) staining following injury with NTS are shown with no difference seen with respect to tubulointerstitial and glomerular mesangial injury in wild-type vs β_2_-AR knockout mice. Scale bars = 50 μm

β_2_-AR flox/flox mice (C57B/6 J background) were obtained from the University of Arizona. To generate podocyte specific β_2_-AR knockout mice, the β_2_-ARflox/flox mice were crossed with podocin-cre (B6.Cg-Tg (NPHS2-cre) 295Lbh/J) mice. The strategy of β_2_-ARflox/flox mouse generation was described previously [9, 17]. Presence of the β_2_-AR-flox gene was confirmed through genotyping using specific primers (Fig. 1B) [18]. Wild-type mice revealed a band of ~ 500 bp, whereas, knockout mice had a band of ~ 550 bp and heterozygous mice showed both bands [18]. Glomeruli were isolated using the magnetic beads-based method as described previously [19, 20]. Total RNA was isolated and β_2_-AR expression in wild-type and β_2_-AR knockout mice glomeruli was evaluated by qPCR using specific primers, forward primer: ACT CAG GAA CGG GAC GAA and reverse primer GCA CAC GCC AAG GAG ATT AT, whereas the rps13 primers have been described previously [15].

### Mouse models of glomerular injury

**Fig. 2:**
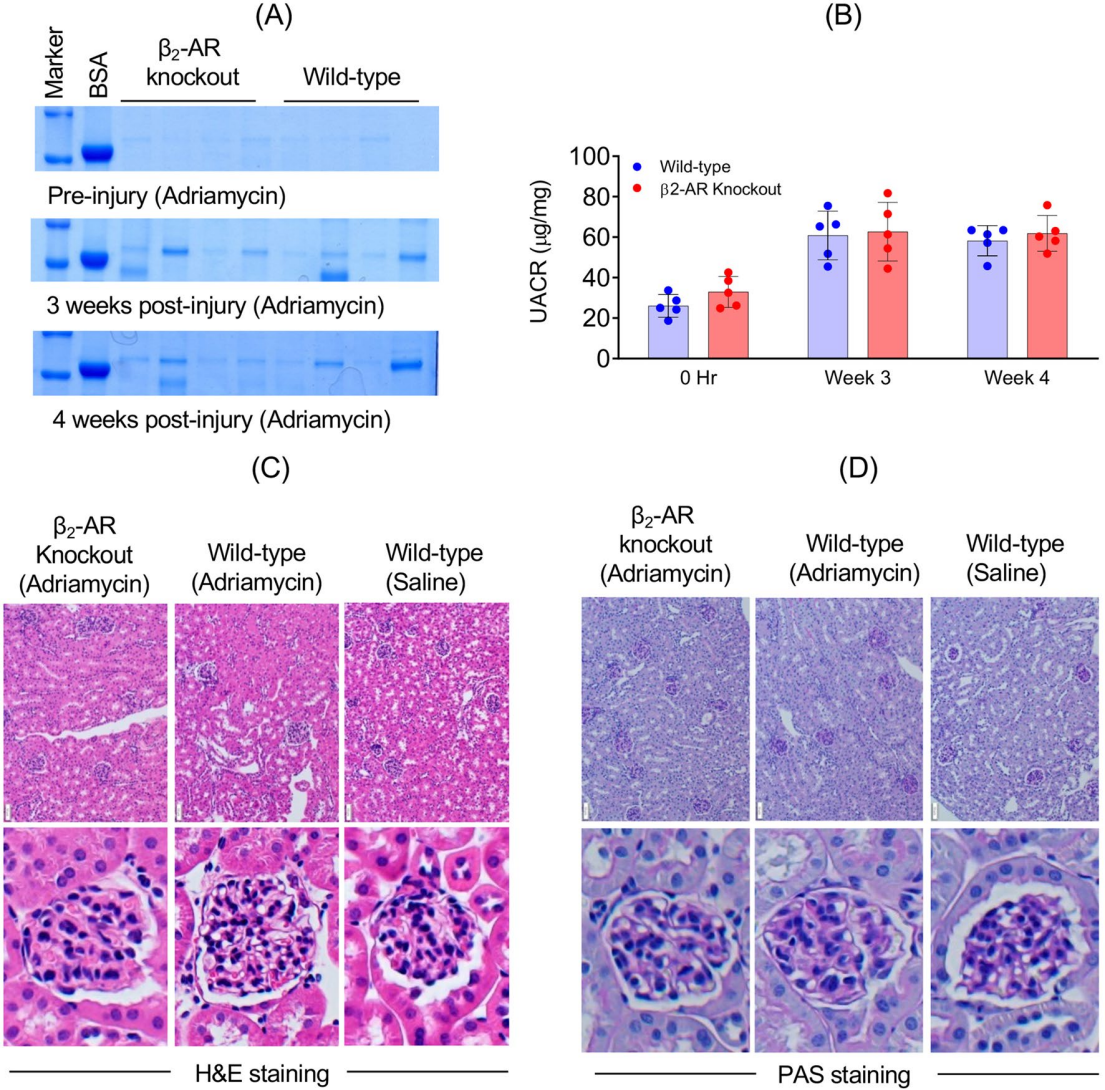
Podocyte-specific deletion of the β_2_-AR in mice does not change their susceptibility to adriamycin: **A** SDS-PAGE analysis of urine samples showed no significant difference in albuminuria pre or post-ADR-injury between wild-type and β_2_-AR knockout mice. **B** The urine albumin/creatinine ratios also showed no significant difference between wild-type and β_2_-AR knockout mice. Data are presented in mean ± SEM and were analyzed using a two-way ANOVA (main effect time: F_2,16_ = 34.65, p < 0.0001; main effect β_2_-AR knockout: F_1,8_ = 1.275, p = 0.2916; interaction: F_2,16_ = 0.1695, p = 0.8456) with a Holm-Sidak adjustment for p values and showed no differences between wild-type and β_2_-AR knockout mice at each timepoint. **C&D** Representative histological sections of the kidneys by hematoxylin and eosin **H&E** and periodic acid-Schiff (PAS) staining are shown with no differences with respect to tubulointerstitial and glomerular mesangial injury seen in the wild-type vs β_2_-AR knockout mice. Scale bars = 50 μm

10–12 week old male β_2_-AR knockout mice (β_2_-AR^fl/fl^/PodCre^+/−^) and their male wild-type (β_2_-AR^fl/fl^/β_2_-AR^+/+^PodCre^+/−^) littermates were treated with 80 µl NTS (Probetex INC, catalog # PTX-001), which induced consistent proteinuria as reported earlier [15, 19]. Urine samples from individual mice were collected at pre-injection, day 1, day 7 and day 14 post-NTS injection. Control vehicle or formoterol (1 mg/kg body weight) were administered intraperitoneally 4 h (h) post NTS-injection, when proteinuria was established. Formoterol injections were repeated every 24 h for 13 days. Detailed experimental plans for NTS-induced glomerular injury, urine collection and drug administration are presented in the schematic diagram (Fig. 2A). All urine samples were spun at 4000 × g for 5 min and then frozen at − 80 °C for subsequent analysis. For the adriamycin (ADR) model 15 mg/kg ADR (aka doxorubicin hydrochloride) (Tocris Bioscience, Cat. No. 2252) was injected in the β_2_-AR knockout mice and their wild-type littermates and proteinuria was evaluated as described previously [15].

### Urinalysis

Urine samples (2.5–5.0 μl) were diluted fivefold with sterile water and analyzed by 10% SDS-PAGE followed by Coomassie blue (CB) staining. The urine albumin/creatinine ratios (UACR) were analyzed by albumin ELISA using Albuwell kit (Ethos Biosciences, Exocell, Product #1011) and creatinine analysis was performed using the endpoint assay (TECO Diagnostics Catalog #C515480) as described previously [15].

### Histological analysis

Mice were perfused with Hanks buffered salt solution (HBSS) and their kidneys isolated at day 14 post-NTS injury and subsequent formoterol treatment. Isolated kidneys were fixed for 12 h in 4% paraformaldehyde (PFA), stored in 70% ethanol and submitted to the MUSC Histology Core for paraffin embedding and sectioning. Sections (5 μm) were cut, deparaffinized and stained with hematoxylin and eosin (H&E), periodic acid-Schiff (PAS) and Masson’s Trichrome as described previously [15, 19]. Histological images were collected on an inverted Zeiss Axiovert-200-M microscope at the Cell & Molecular Imaging core facility of the MUSC. The scoring of histological stained sections was performed blindly as described earlier [15, 19]. Briefly, glomerulosclerosis severity scoring was performed using a subjective scale, as follows: 1 = none/trace, 2 = mild/segmental, 3 = moderate/global, and 4 = severe sclerosis. About 40–50 glomeruli from each mouse and n = 5 mice was used in each group.

The immunostaining of mouse kidney sections was performed as described previously [10]. Briefly, kidney sections were immunostained using specific primary antibodies for NEPH1, a member of the nephrin-like protein family and a component of the slit diaphragm, that we previously generated, SYNAPTOPODIN (Abcam, Catalog # ab224491) and α-SMA (Santa Cruz, Catalog # c53142), followed by Alexa Fluor-labeled secondary antibodies and 4*′*,6-diamidino-2-phenylindole (DAPI) (all ThermoFisher Scientific). Single plane confocal images were collected using Olympus FV1200 MPE confocal microscope fitted with 60X oil objective at the MUSC microscopy core facility. Image acquisition parameters were kept constant throughout the experiment. Mean pixel intensity estimation and Pearson’s correlation coefficient analysis for colocalization was performed using Image-J software as described previously [15].

### Statistical analyses

Data are presented in mean ± SEM. The Student’s t test or one-way ANOVA with Tukey’s HSD or two-way ANOVA with a Holm-Sidak adjustment for p values were performed using the GraphPad Prism 8 software. A p value of  ≤  0.05 was considered as statistically significant.

## Results

### Injury to podocytes upregulates β_2_-AR expression

We hypothesized that injury to podocytes may affect β_2_-AR expression levels. The expression profile of the β_2_-AR in RNA-seq data from various podocyte injury models including adriamycin (ADR) and puromycin aminonucleoside (PAN, GEO# GSE124622 & GSE117669) was, therefore, determined [15, 16]. The results showed upregulation of the β_2_-AR gene (adjusted p values, < 0.05, Fig. 1A), which is consistent with increased mitochondrial biogenesis (MB) in response to injury and may serve as an adaptive response that podocytes use to meet the increased energy demand during recovery.

### Genetic deletion of β_2_-AR in mouse podocytes

To evaluate the in vivo significance of the β_2_-AR in podocytes, we first generated podocyte-specific β_2_-AR knockout mice (β_2_-AR^fl/fl^/PodCre^+/−^) by crossing the β_2_-AR floxed (β_2_-AR^fl/+^) mice with PodCre^±^ mice. The control littermates (wild-type mice) were the following genotypes: β_2_-AR^+/+^PodCre^+/−^, β_2_-AR^fl/fl^, or β_2_-AR^+/+^. The genotype of the experimental mice was β_2_-AR^fl/fl^PodCre^+/−^ (Fig. 1B). Genotyping results confirmed the presence of the β_2_-AR floxed gene (Fig. 1C). qPCR analysis of glomeruli isolated from wild-type and β_2_-AR knockout mice showed significant reduction of β_2_-AR mRNA in the glomeruli of β_2_-AR knockout vs. wild-type mice (t_3.31_ =  − 3.8068, p = 0.02684, Fig. 1D). The presence of some β_2_-AR mRNA in the glomeruli of β_2_-AR knockout mice likely represents β_2_-AR mRNA from non-podocyte glomerular cells.

### Podocyte-specific β_2_-AR genetic deletion in mice does not affect their susceptibility to glomerular injury

The β_2_-AR knockout mice developed normally and did not show any signs of albuminuria or glomerular injury. This led us to investigate whether the loss of the β_2_-AR in mice podocytes would change their susceptibility to injury using an acute glomerular injury model, nephrotoxic serum (NTS, Fig. 1E). A two-way ANOVA of urine from the NTS-injured mice by sodium dodecyl-sulfate polyacrylamide gel electrophoresis (SDS-PAGE) and albumin/creatinine ratio (UACR) showed no significant difference in the injury level between wild-type and β_2_-AR knockout mice over time (main effect β_2_-AR knockout: F_1,8_ = 0.8453, p = 0.5716; interaction β_2_-AR knockout and time: F_2,16_ = 0.6400, p = 0.5403) (Fig. 1F,G and Supplemental Figs. 1 and 2). Furthermore, histological review of the kidney sections using hematoxylin and eosin (H&E) staining showed no histological differences with respect to tubulointerstitial and glomerular mesangial injury between wild-type and β_2_-AR knockout mice following NTS injury (Fig. 1H).

Because it has been shown that genetic deletion of pathogenic genes such as *Myh9* in mice that are raised on C57B/6 J background may change their susceptibility to ADR [10], we tested whether the loss of β_2_-AR in podocytes similarly affected their susceptibility to ADR-induced injury. Wild-type and β_2_-AR knockout mice were treated with ADR and the urine was analyzed. The wild-type and β_2_-AR knockout mice had similar levels of ADR-induced injury over time (main effect β_2_-AR knockout: F_1,8_ = 1.275, p = 0.2916; interaction: F_2,16_ = 0.1695, p = 0.8456) though the injury was less severe than with NTS (Fig. 2 and Supplemental Figs. 2 and 3).

### β_2_-AR knockout mice show impaired β_2_-AR agonist-induced recovery

**Fig. 3:**
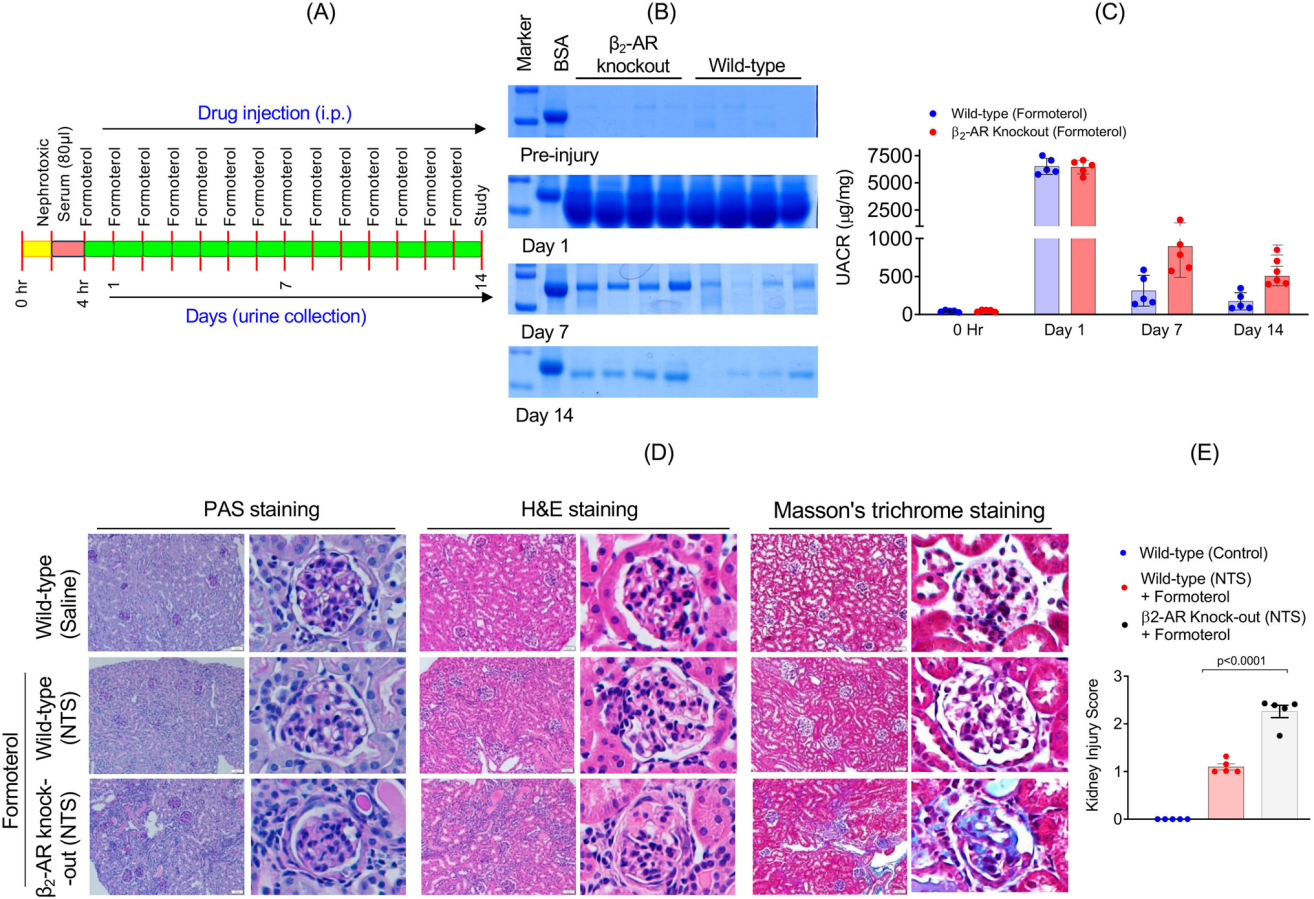
β_2_-AR knockout mice show impaired formoterol-induced recovery in mice: **A** Schematic of the experimental plan. **B** Urine samples were evaluated by SDS-PAGE and **C** urine albumin/creatinine ratios (UACR) were measured which showed a reduction in albuminuria at day 7 in wild-type but not in β_2_-AR knockout mice treated with formoterol. n = 5 mice per group. Data are presented as mean ± SEM. Data were analyzed using a two-way ANOVA (main effect time: F_3,24_ = 731.17, p < 0.0001; main effect β_2_-AR knockout F_1,8_ = 2.191, p = 0.1774; interaction: F_3,24_ = 1.7822, p = 0.1789) with a Holm-Sidak adjustment for p values. **D** Representative kidney sections from mice sacrificed at day 14 post NTS injection indicated that wild-type mice treated with formoterol had a higher number of normal glomeruli with reduced focal atrophy, proteinaceous tubular casts and tubular dilation compared to β_2_-AR knockout mice treated with formoterol that had increased sclerotic glomeruli, fibrosis, and tubular casts. Scale bars = 50 μm. **E** The kidney injury score was calculated and showed reduced kidney injury in wild-type mice compared to the β_2_-AR knockout mice treated with formoterol. PAS and H&E sections were subjected to blinded scoring of renal injury using a scale, as follows: 1 = none, 2 = mild glomerulosclerosis, 3 = moderate glomerulosclerosis, and 4 = severe glomerulosclerosis. n = 5 mice in each group. These data were analyzed using a one-way ANOVA with Tukey’s HSD (F_2,12_ = 186.6, p < 0.0001)

We previously showed that the β_2_-AR agonist formoterol promoted recovery from NTS- and ADR-induced glomerular injury [15]. Because the injury was greater with NTS than with ADR, we next determined whether signaling through the β_2_-AR was responsible for the formoterol-induced recovery from glomerular injury. Wild-type and β_2_-AR knockout mice (10–12 weeks old) were treated with NTS (Fig. 3A). Following the establishment of proteinuria at 4 h post NTS injection, the long-acting β_2_-AR agonist formoterol (1 mg/kg) was injected intraperitoneally (IP) in both wild-type and β_2_-AR knockout mice. The 1 mg/kg IP dose was chosen as this is the dose and route of administration that we and others used in previous studies where a rescue effect was demonstrated following acute kidney injury [15]. The urine samples from these mice were evaluated at days 1, 7 and 14 for proteinuria by SDS-PAGE using a 2-way ANOVA (Fig. 3B and Supplemental Fig. 4). Urine albumin/creatinine ratios (UACR) confirmed that albuminuria differed by time post NTS injury and formoterol treatment in both wild-type and β_2_-AR knockout mice (main effect time: F_3,24_ = 731.17, p < 0.0001; main effect β_2_-AR knockout F_1,8_ = 2.191, p = 0.1774; interaction: F_3,24_ = 1.7822, p = 0.1789)_,_ specifically post-hoc analysis using Holm-Sidak adjustment found both groups differed significantly in UACR from day 0 to day 1 (p < 0.0001 both groups). Post-hoc analysis with Tukey’s adjustment showed no differences in UACR between wild-type and β_2_-AR knockout mice at day 0, 1, or 14 (p values not shown). At 7 days there was a difference; however, this difference was not significant after accounting for the four comparisons using Tukey’s HSD (p = 0.0875) (Fig. 3B, C and Supplemental Fig. 5). This suggests that loss of β_2_-AR affects the ability of mice to recover from glomerular injury with formoterol. To further evaluate the histological changes, kidney sections from these mice (sacrificed at 14 days post NTS injection), were analyzed by staining with H&E, periodic acid-Schiff (PAS), and Masson’s trichrome. Consistent with albuminuria, the sections from β_2_-AR knockout mice showed increased tubular dilatation, PAS positive casts, and fibrotic and sclerotic glomeruli compared to the wild-type mice (Fig. 3D). A decreased kidney injury score was also found in wild-type compared to β_2_-AR knockout mice following NTS injury and treatment with formoterol (F_2,12_ = 186.6, p < 0.0001) (Fig. 3E and Supplemental Fig. 5).

### Membrane localization of the slit diaphragm protein NEPH1 was not restored in β_2_-AR knockout mice treated with formoterol

**Fig. 4:**
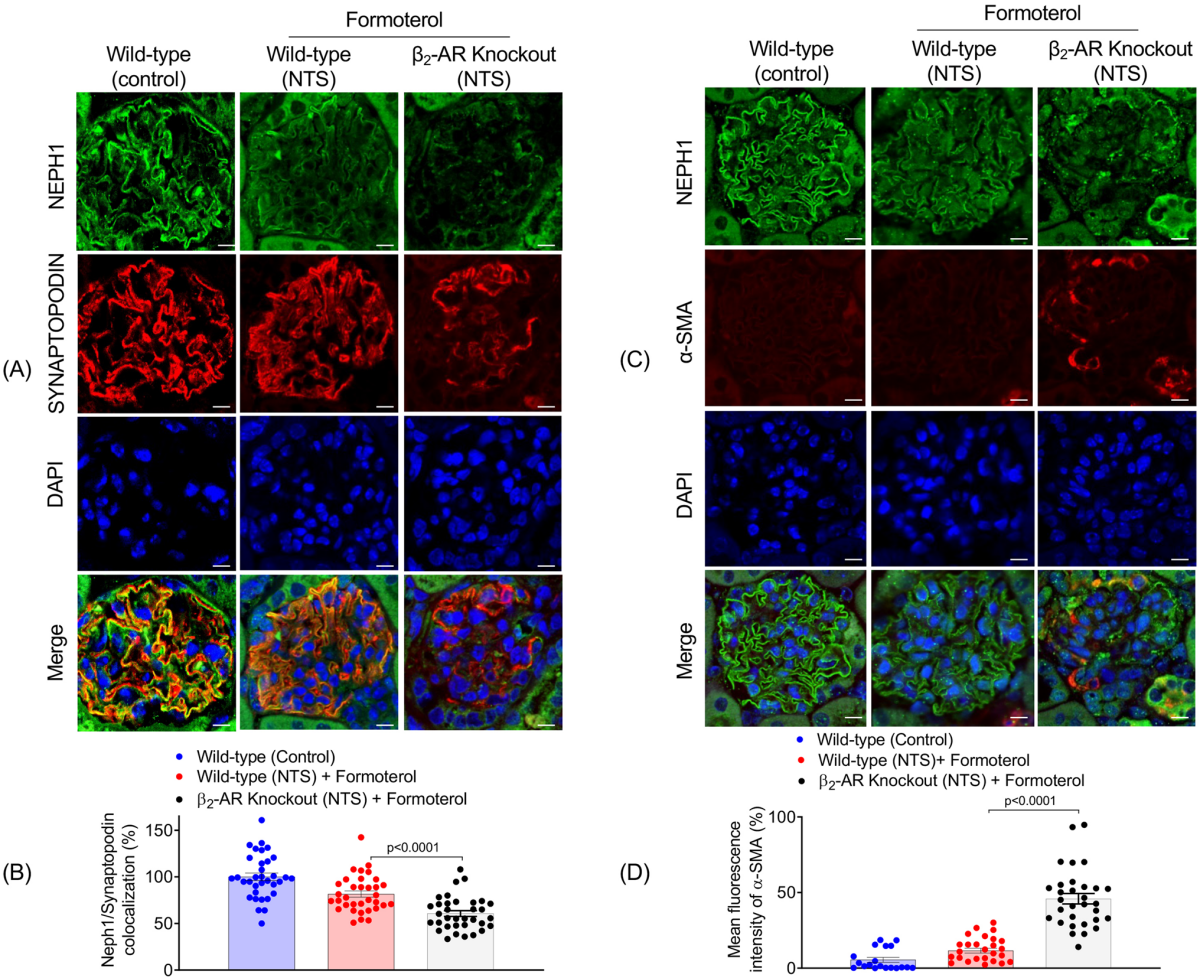
Podocyte cell membrane localization of the slit diaphragm protein NEPH1 is not restored in β_2_-AR knockout mice following NTS-induced injury and treatment with formoterol. **A** Kidney sections of wild-type and β_2_-AR knockout mice were immunostained with NEPH1 (green) and SYNAPTOPODIN (red) antibodies and cell nuclei were counter-stained with DAPI which was present in the mounting medium (blue). NTS-induced loss of NEPH1 was largely restored to the podocyte cell membrane in wild-type, but not β_2_-AR knockout, mice treated with formoterol. As expected, NEPH1 co-localized with SYNAPTOPODIN at the podocyte cell membrane. Scale bars = 20 μm. **B** Analysis showed increased NEPH1 colocalization with SYNAPTOPODIN in wild-type compared to β_2_-AR knockout mice treated with formoterol. Data are presented as mean ± SEM. Data were analyzed using a one-way ANOVA (F_2,99_ = 31.33, p < 0.0001) with Tukey’s HSD. *P* ≤ 0.0001. n = 5 mice in each group. Each dot represents individual glomeruli. **C** Immunostaining of kidney sections using NEPH1 (green) and alpha smooth muscle actin (α-SMA) (red) antibodies along with DAPI in the mounting medium (blue) showed formoterol treatment reduced α-SMA expression, which is an injury marker, in wild-type mice but not in β_2_-AR knockout mice. Scale bars = 20 μm. **D** Quantitative analysis of immunofluorescence images confirmed increased α-SMA expression in formoterol-treated β_2_-AR knockout, compared to wild type, mice. Data are presented as mean ± SEM. Data were analyzed using a one-way ANOVA (F_2,75_ = 69.11, p < 0.0001) with Tukey’s HSD. *P* ≤ 0.0001. n = 5 mice in each group. Each dot represents individual glomeruli

We previously showed that the slit-diaphragm protein NEPH1 mis-localizes and is lost from the podocyte cell membrane in response to injury, but re-localizes during recovery from injury [15, 21, 22]. To further evaluate the ability of β_2_-AR knockout mice to recover from injury, kidney sections from wild-type and β_2_-AR knockout mice injured with NTS and treated with formoterol were immunostained with NEPH1 and SYNAPTOPODIN antibodies. While NTS-induced injury resulted in loss of NEPH1 from the podocyte cell membrane, formoterol treatment restored NEPH1 localization to the podocyte cell membrane. As expected, there was increased colocalization of NEPH1 with SYNAPTOPODIN in wild-type mice but not in β_2_-AR knockout mice (Fig. 4A). Quantitation confirmed increased NEPH1 colocalization with SYNAPTOPODIN in the glomeruli of wild-type compared to β_2_-AR knockout mice injured with NTS and treated with formoterol (F_2,99_ = 31.33, p < 0.0001) (Fig. 4B and Supplemental Fig. 6). These data indicate that formoterol signaling through the β_2_-AR is required for the functional and structural recovery of podocytes from injury.

### β_2_-AR knockout, compared to wild-type, mice treated with formoterol have increased fibrosis following NTS-induced injury

Alpha smooth muscle actin (α-SMA) is highly expressed in the presence of renal fibrosis and glomerulosclerosis [23–25]. Since NTS treatment is known to induce glomerulosclerosis [15, 19], we evaluated α-SMA expression in kidney sections from NTS-injured wild-type and β_2_-AR knockout mice treated with formoterol. Expression of α-SMA was significantly downregulated in wild-type compared to β_2_-AR knockout mice injured with NTS and treated with formoterol (F_2,75_ = 69.11, p < 0.0001) (Fig. 4C, D and Supplemental Fig 6). We observed partial changes in the expression pattern in many glomeruli due to the sclerotic phenotype. Collectively, these results support the conclusion that the β_2_-AR is required for formoterol-induced podocyte recovery from injury.

## Discussion

Podocytes are a critical component of the glomerular filtration system and are the primary targets in the majority of glomerular diseases [26–28]. Podocyte dysfunction and apoptosis is associated with loss of renal function leading to end-stage kidney disease (ESKD) [28, 29]. Thus, podocytes are a key therapeutic target for preventing damage to the glomerular filtration system and restoring renal function following injury. We recently reported that formoterol treatment restored podocyte function following glomerular injury [15]. In this study, using podocyte-specific β_2_-AR knockout mice, we provide experimental evidence that the β_2_-AR in podocytes is required for recovery of renal function. Although β_2_-AR is expressed in podocytes [8, 15], little is known about its significance in podocyte biology. Our recent study demonstrated that mitochondrial biogenesis (MB), which allows podocytes to meet the metabolic and energy requirements during injury or disease conditions, was increased in response to treatment with formoterol [8]. Mechanistically, this increase in podocytes was attributed to the induction of PGC-1α (a transcriptional co-activator of MB) and key components of the mitochondrial electron transport chain (ETC) [15].

Further evidence for the involvement of the β_2_-AR in podocyte repair came from our mRNA expression analysis which showed that β_2_-AR expression was increased in in vivo models of podocyte injury (Fig. 1). Using β_2_-AR knockout mice we provide genetic evidence that the β_2_-AR is required for podocyte repair following formoterol treatment. While the β_2_-AR knockout mice responded similarly to wild-type mice in the face of injury, their recovery in response to formoterol was significantly attenuated. Collectively, these results support a critical role for the podocyte β_2_-AR in recovery of renal function following injury. Additionally, our results suggest that the renoprotective effects of formoterol are not due to off target effects, but instead are due to the specific activation of β_2_-AR in podocytes.

**Fig. 5:**
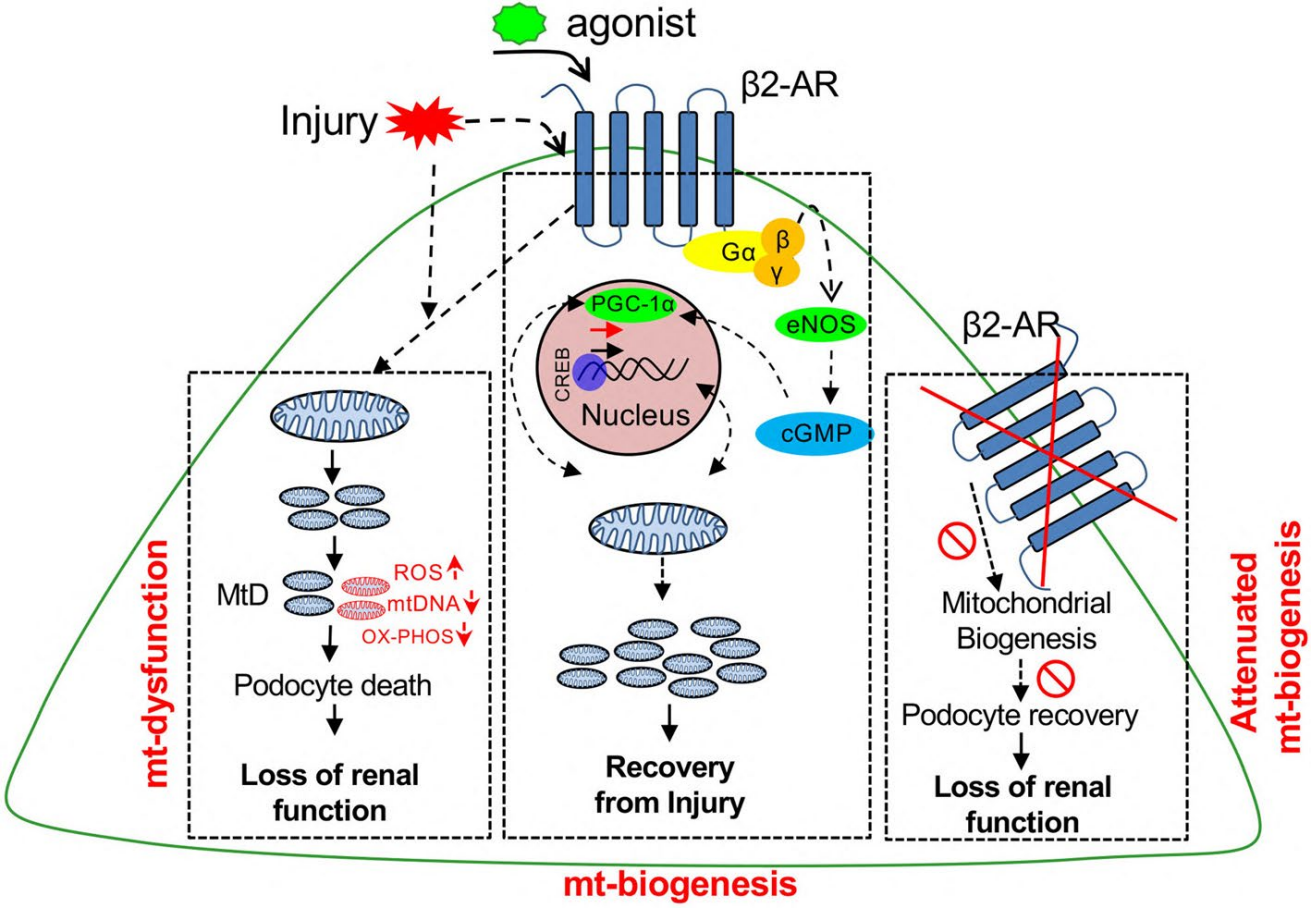
Schematic of the role of the β2-AR in podocyte repair following injury. Mitochondrial (mt) dysfunction due to podocyte injury leads to cell death and loss of renal function. Pharmacological stimulation of the β2-AR induces mt-biogenesis leading to enhanced recovery of podocytes from injury. Deletion of the β2-AR from podocytes significantly attenuates the recovery response in podocytes. ROS = reactive oxygen species. Gα,β,γ  = components of G-protein subunit of the β2-AR involved in signaling. eNOS = endothelial nitric oxide synthase. cGMP = cyclic guanosine monophosphate. CREB = cAMP response element-binding protein. PGC1α = peroxisome proliferator-activated receptor-gamma coactivator 1 alpha, a member of a family of transcription coactivators that plays a central role in the regulation of cellular energy metabolism

In conclusion, while our previous study showed that pharmacological activation of β_2_-AR accelerated recovery of glomerular function by reducing proteinuria and ameliorating kidney pathology, the present investigation revealed that this recovery is mediated by the β_2_-AR in podocytes (Fig. 5). Thus, therapies specifically targeting the podocyte β_2_-AR may be of therapeutic value.

### Supplementary Information

Below is the link to the electronic supplementary material.

Supplementary file1 (JPG 710 KB)

Supplementary file2 (JPG 485 KB)

Supplementary file3 (JPG 813 KB)

Supplementary file4 (JPG 318 KB)

Supplementary file5 (JPG 475 KB)

Supplementary file6 (JPG 385 KB)

Supplementary file7 (DOCX 1618 KB)

## Data Availability

All datasets generated for this study are included in the article. All the raw data will be provided upon reasonable request.

## Abbreviations

ADR: Adriamycin
ANOVA: Analysis of variance
α-SMA: Alpha smooth muscle actin
β_2_-AR: Beta2 adrenergic receptor
β_2_-AR^fl/fl^/PodCre: Podocyte-specific β_2_-AR knockout mice
B6.Cg-Tg(NPHS2-cre)295Lbh/J: Podocin Cre mice
cGMP: Cyclic guanosine monophosphate
CREB: CAMP response element-binding protein
DAPI: 4′,6-Diamidino-2-phenylindole
eNOS: Endothelial nitric oxide synthase
ESKD: End-stage kidney disease
ETC: Electron transport chain
FSGS: Focal and segmental glomerulosclerosis
Gα,β,γ: Components of G-protein subunit of the β2-AR involved in signaling
GBM: Glomerular basement membrane
GPCR: G-protein-coupled receptor
H&E: Hematoxylin and eosin
IACUC: Institutional Animal Care and Use Committee
IP: Intraperitoneal
MB: Mitochondrial biogenesis
Mt: Mitochondria
NTS: Nephrotoxic serum
PAS: Periodic acid-Schiff
PGC1α: Peroxisome proliferator-activated receptor-gamma coactivator 1 alpha
PFA: Paraformaldehyde
PAN: Puromycin aminonucleoside
ROS: Reactive oxygen species
SDS-PAGE: Sodium dodecyl-sulfate polyacrylamide gel electrophoresis
SEM: Standard error of mean
UACR: Urine albumin/creatinine ratios
