# Supplementary figures and images for: Role of the β2-adrenergic receptor in podocyte injury and recovery
Source: Pharmacol Rep. 2024 Apr 26;76(3):612–21. doi: 10.1007/s43440-024-00594-5 (PMC11126448; doi:10.1007/s43440-024-00594-5)

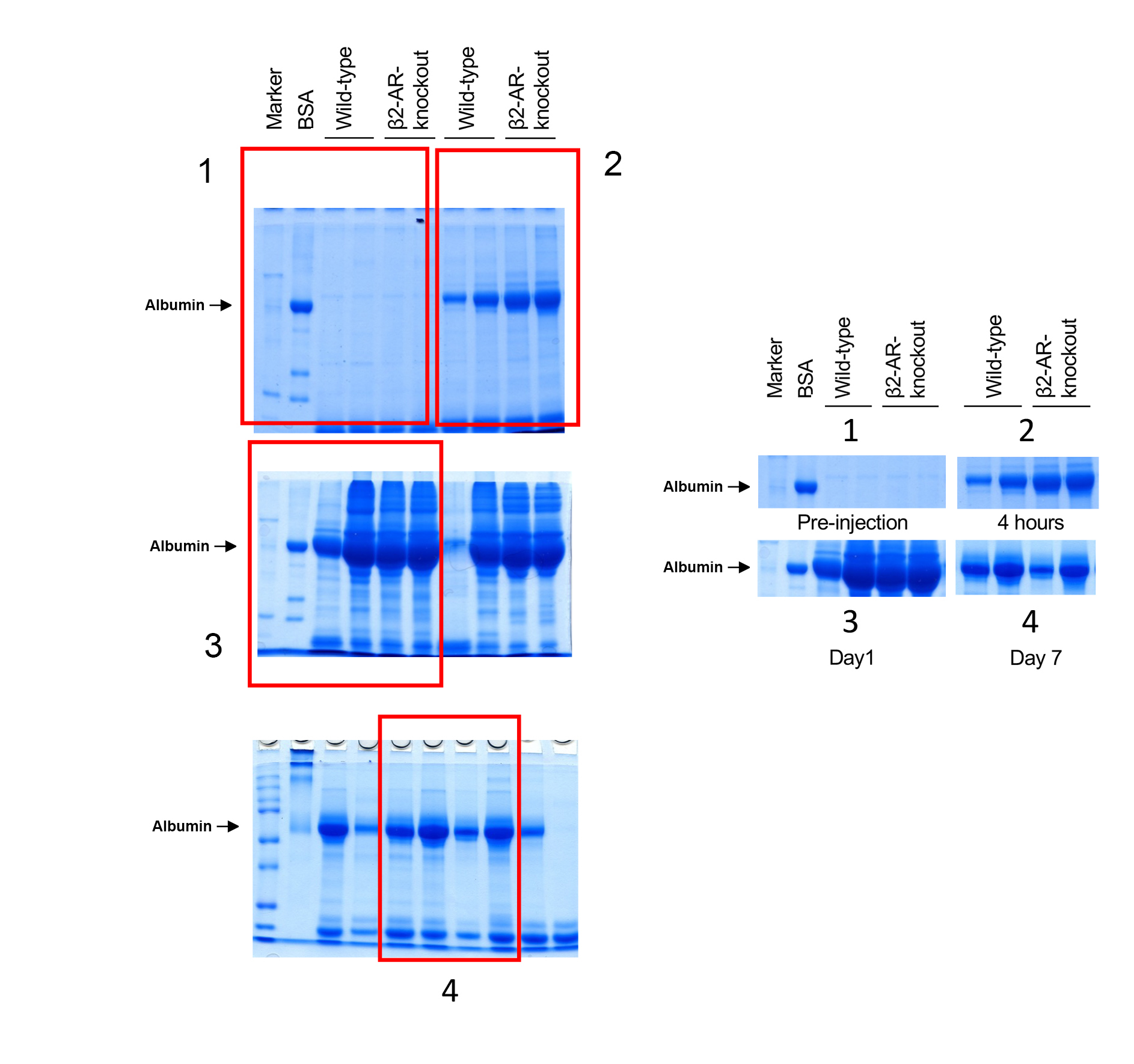

Supplement: Supplementary file 1 — Supplementary file1 (JPG 710 KB) [file 43440_2024_594_MOESM1_ESM.jpg]

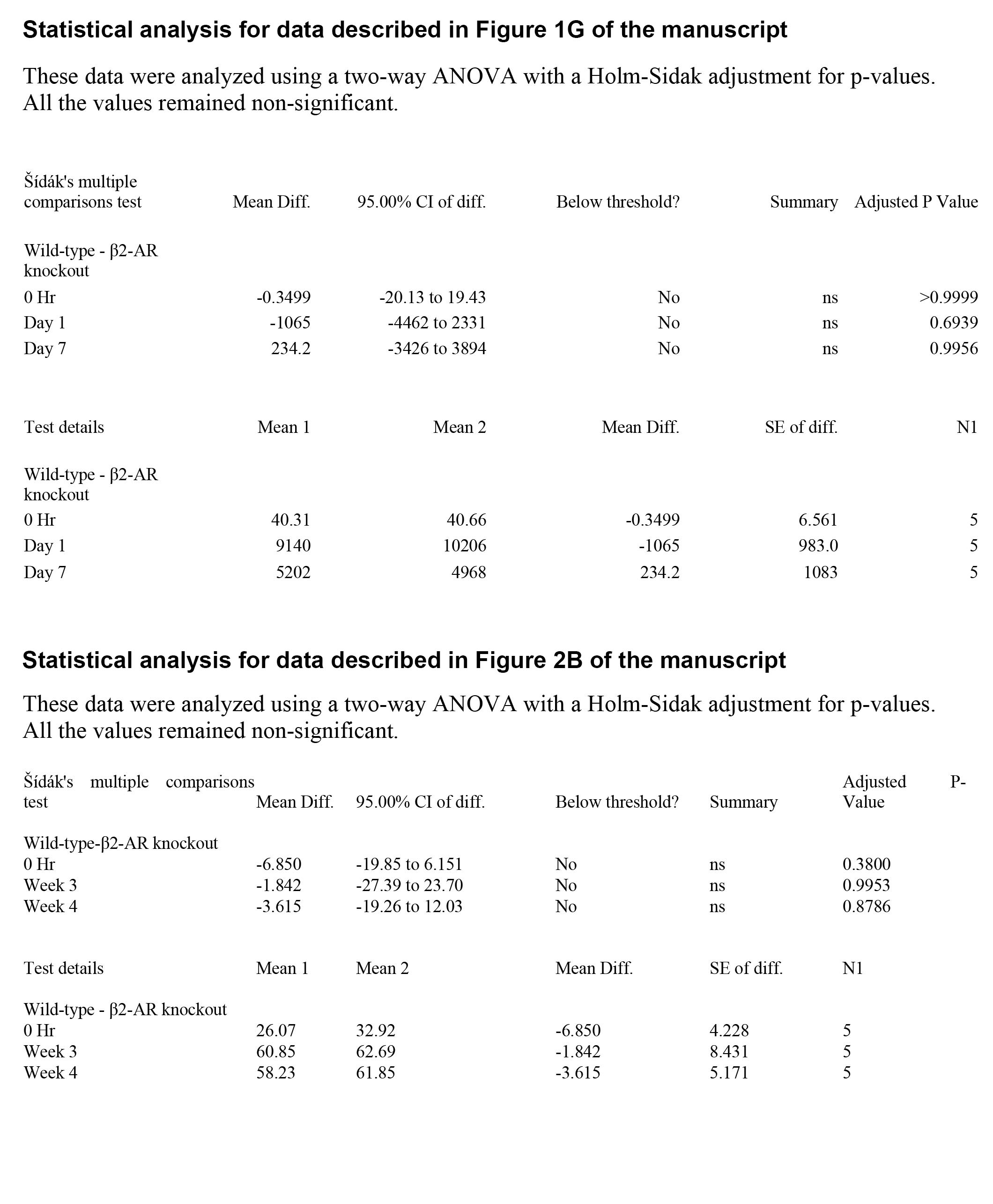

Supplement: Supplementary file 2 — Supplementary file2 (JPG 485 KB) [file 43440_2024_594_MOESM2_ESM.jpg]

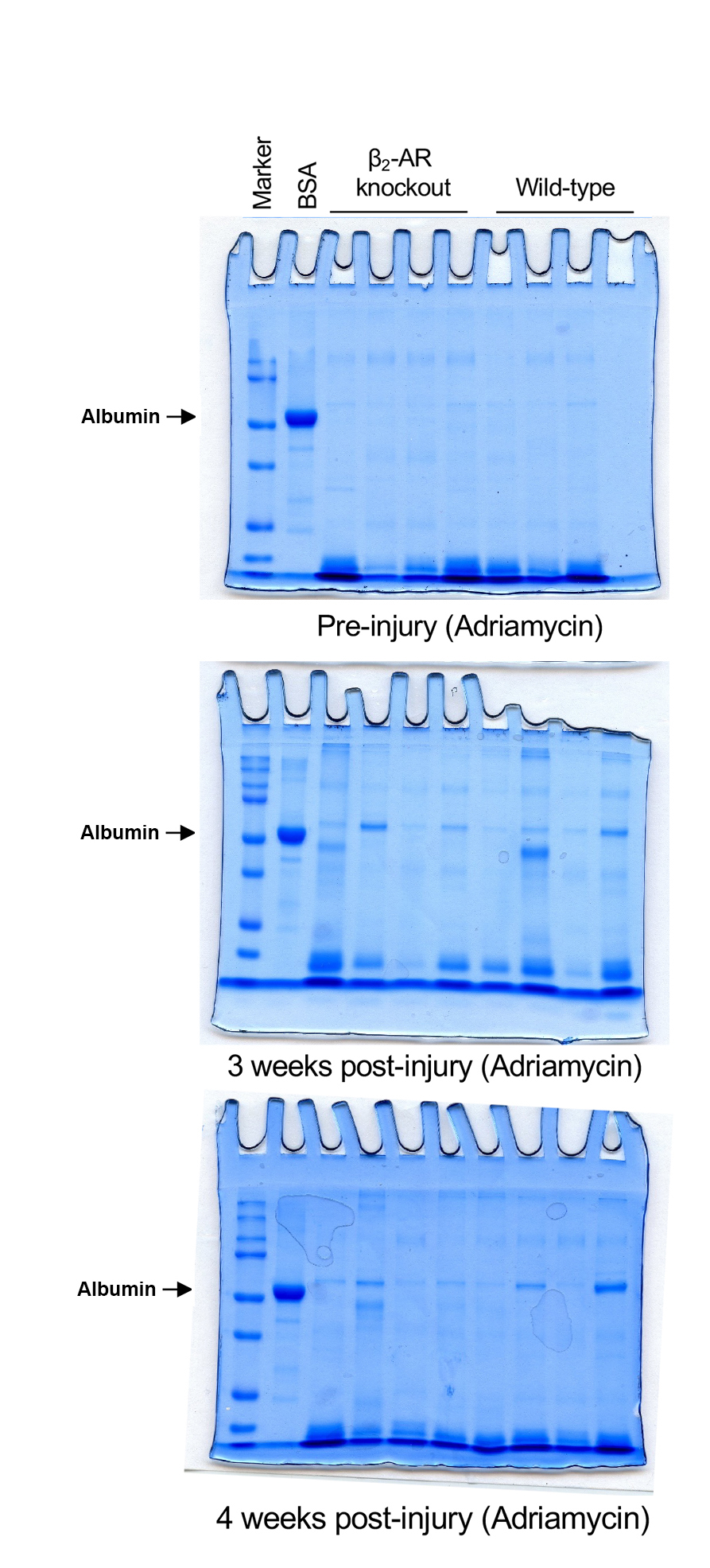

Supplement: Supplementary file 3 — Supplementary file3 (JPG 813 KB) [file 43440_2024_594_MOESM3_ESM.jpg]

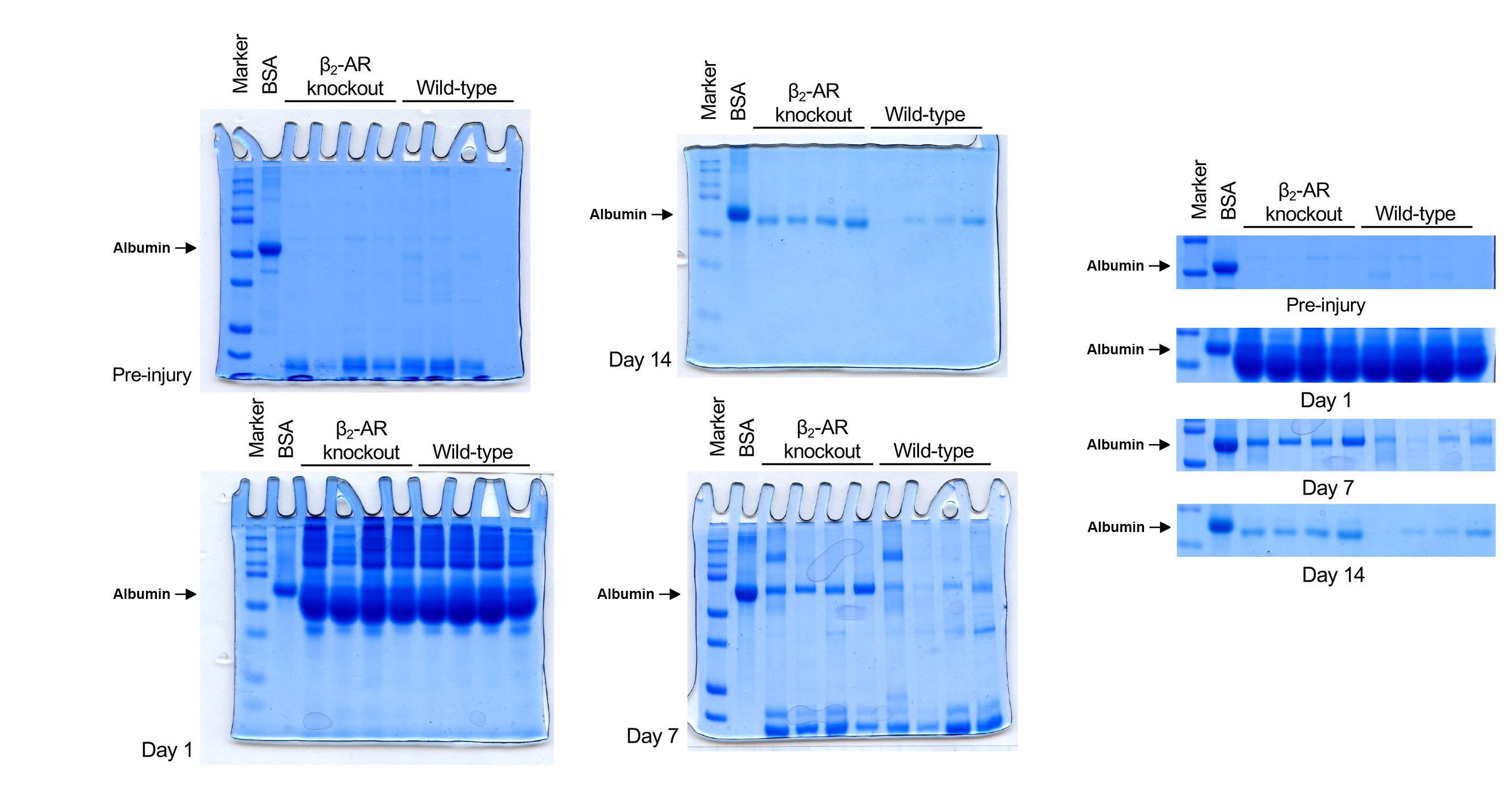

Supplement: Supplementary file 4 — Supplementary file4 (JPG 318 KB) [file 43440_2024_594_MOESM4_ESM.jpg]

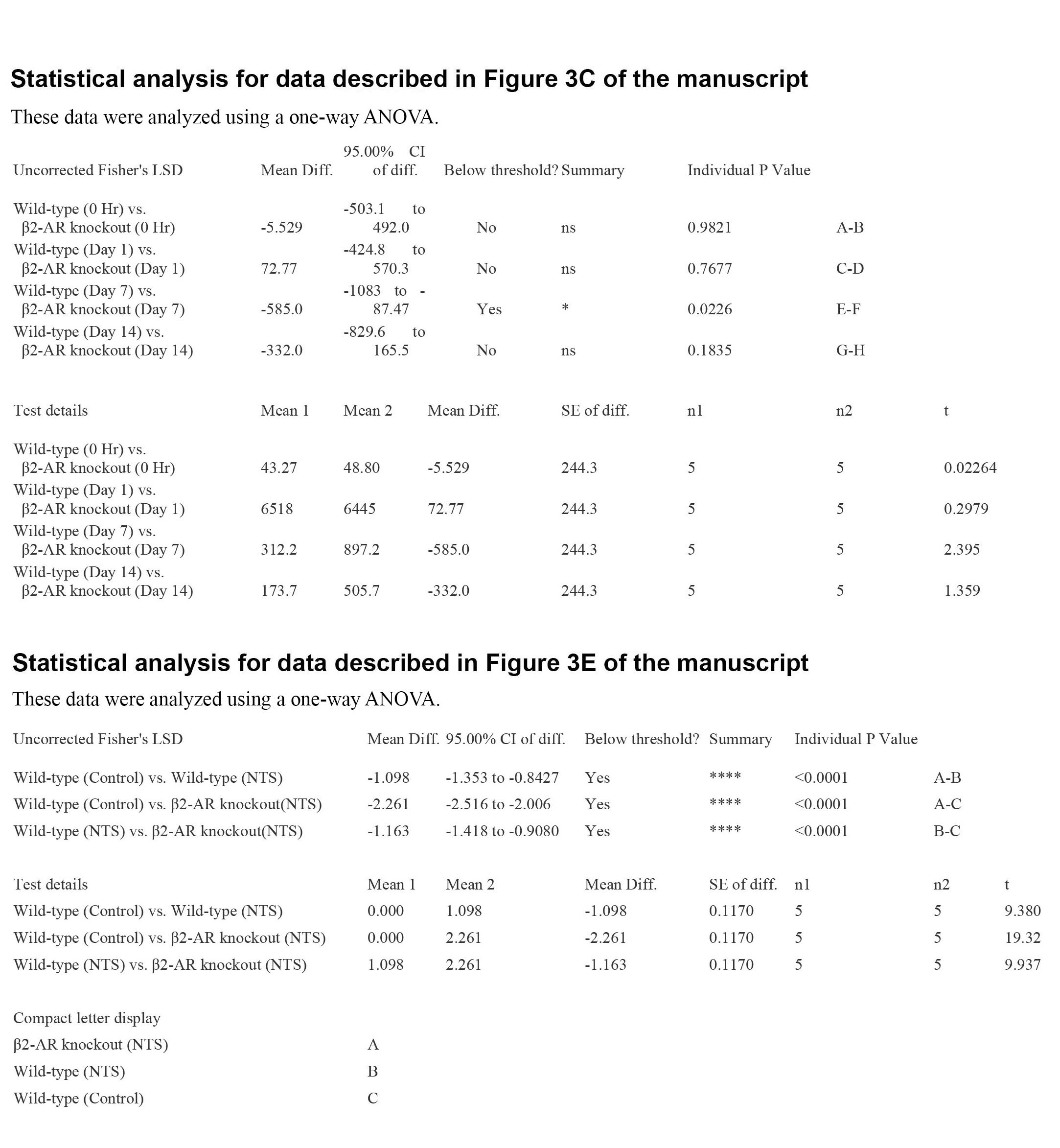

Supplement: Supplementary file 5 — Supplementary file5 (JPG 475 KB) [file 43440_2024_594_MOESM5_ESM.jpg]

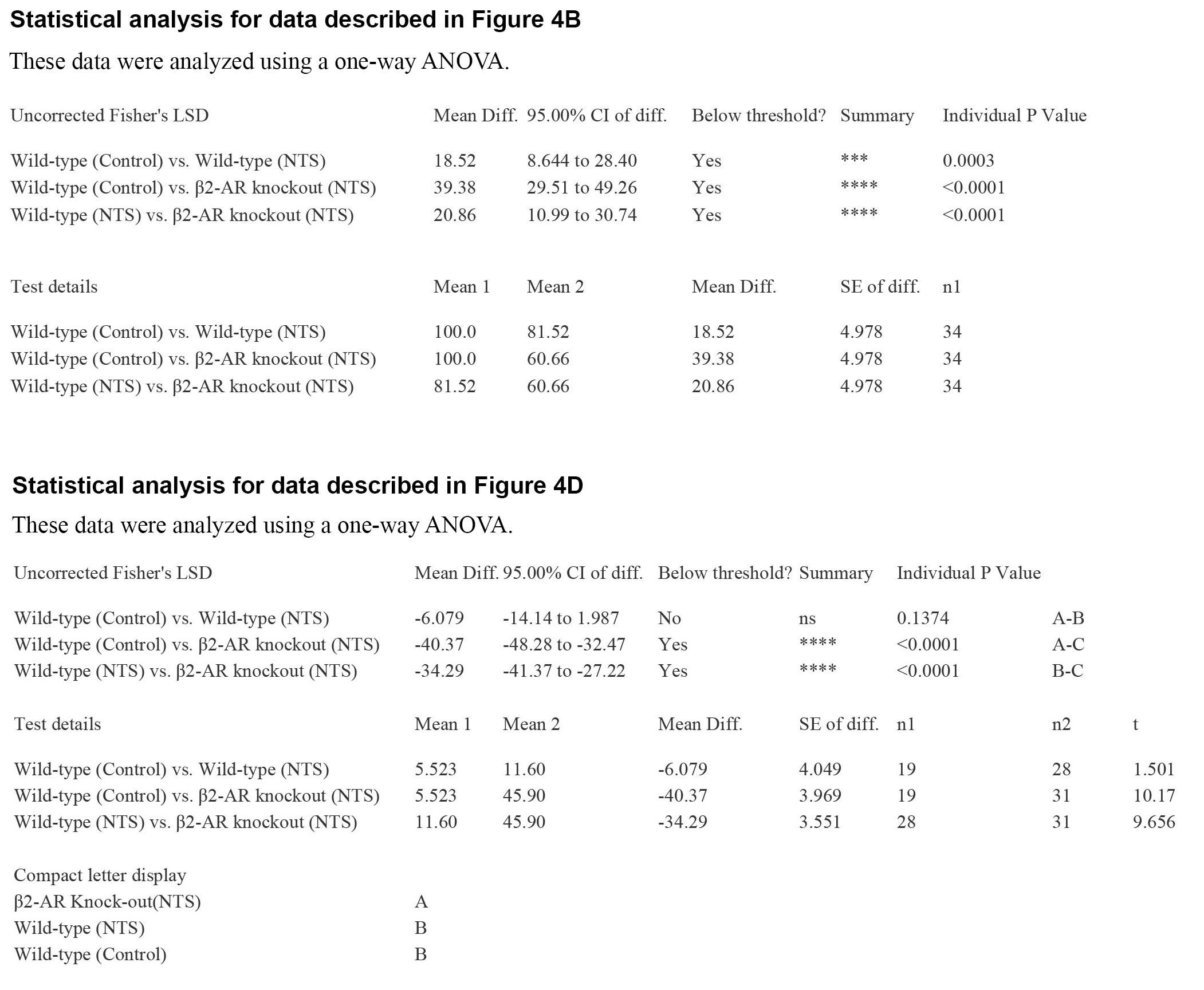

Supplement: Supplementary file 6 — Supplementary file6 (JPG 385 KB) [file 43440_2024_594_MOESM6_ESM.jpg]
