# Supplementary material for: Role of the β2-adrenergic receptor in podocyte injury and recovery
Source: Pharmacol Rep. 2024 Apr 26;76(3):612–21. doi: 10.1007/s43440-024-00594-5 (PMC11126448; doi:10.1007/s43440-024-00594-5)
Supplement: Supplementary file 7 — Supplementary file7 (DOCX 1618 KB) [file 43440_2024_594_MOESM7_ESM.docx]

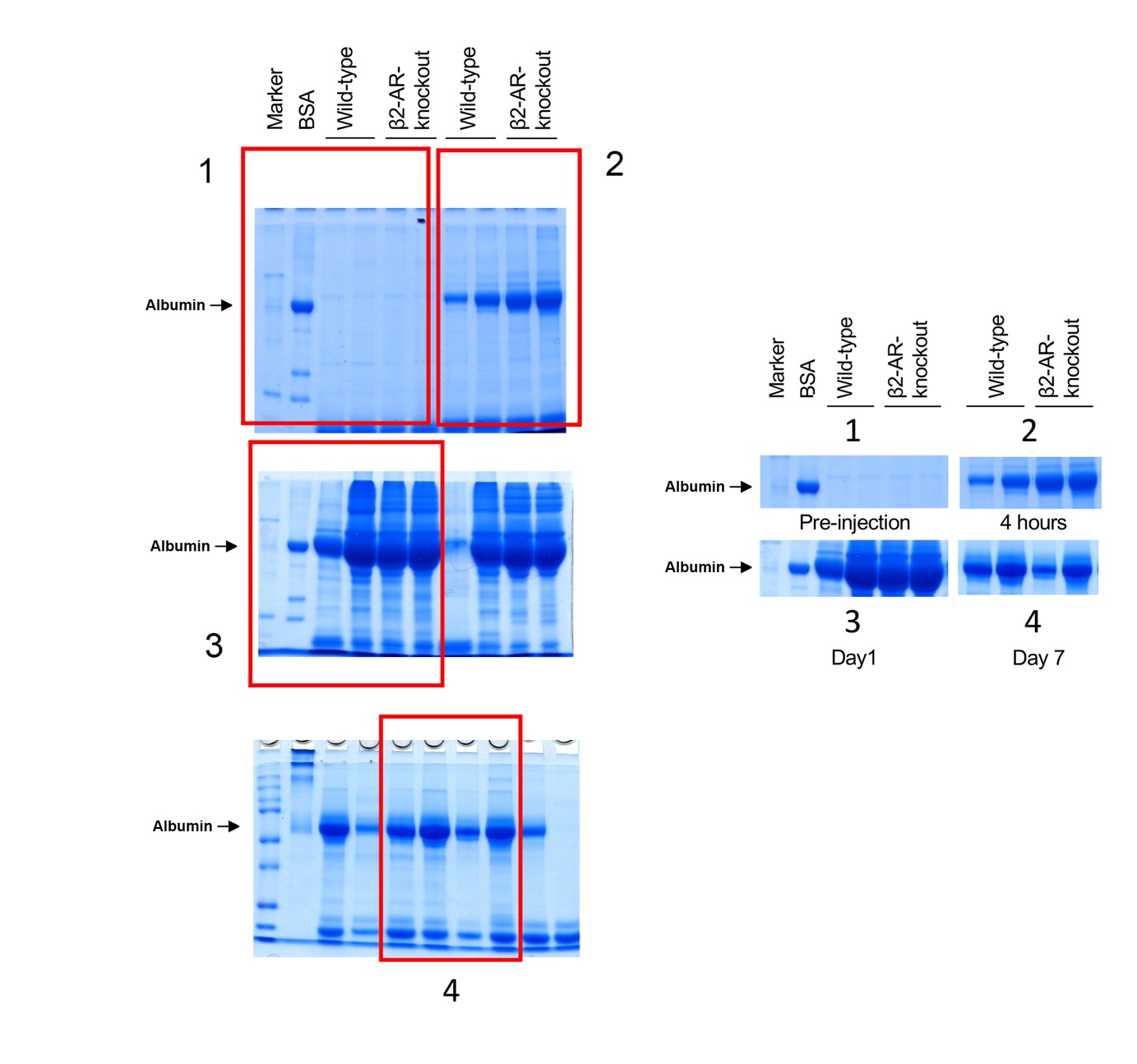


**Supplemental Figure 1: Whole image of Coomassie blue stained gels.** The whole gel images correspond to the data presented in Figure 1F.

**
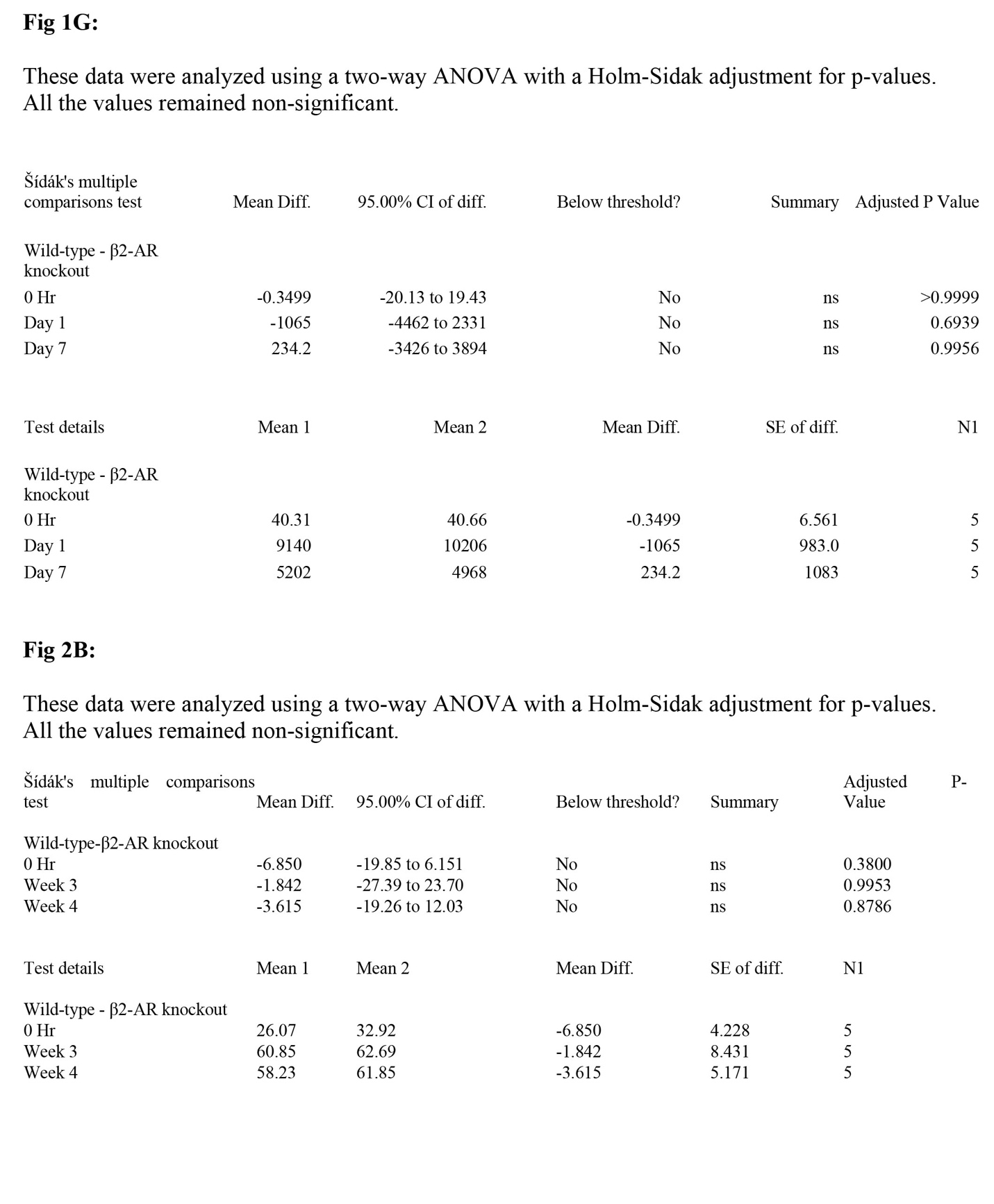
**

**Supplemental Figure 2: Statistical analyses for Figures 1G and 2B.**


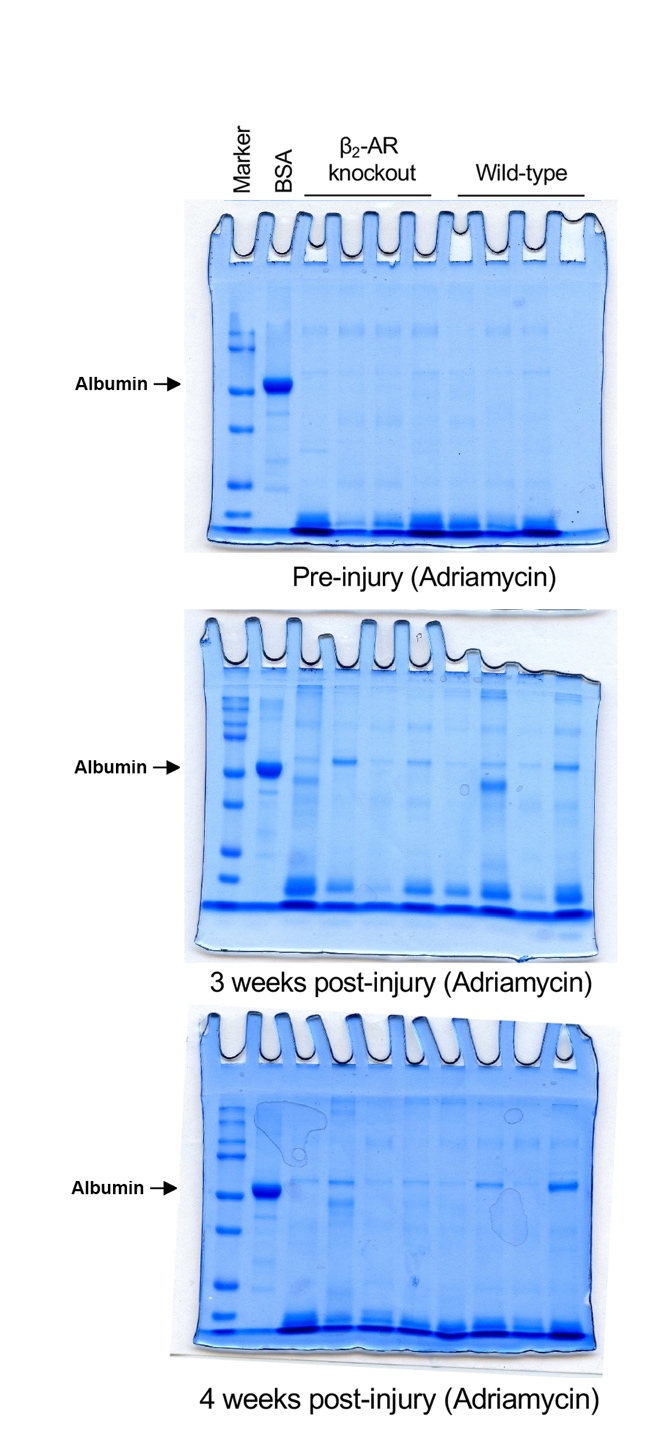


**Supplemental Figure 3: Whole image of Coomassie blue stained gels.** The whole gel images correspond to the data presented in Figure 2A.


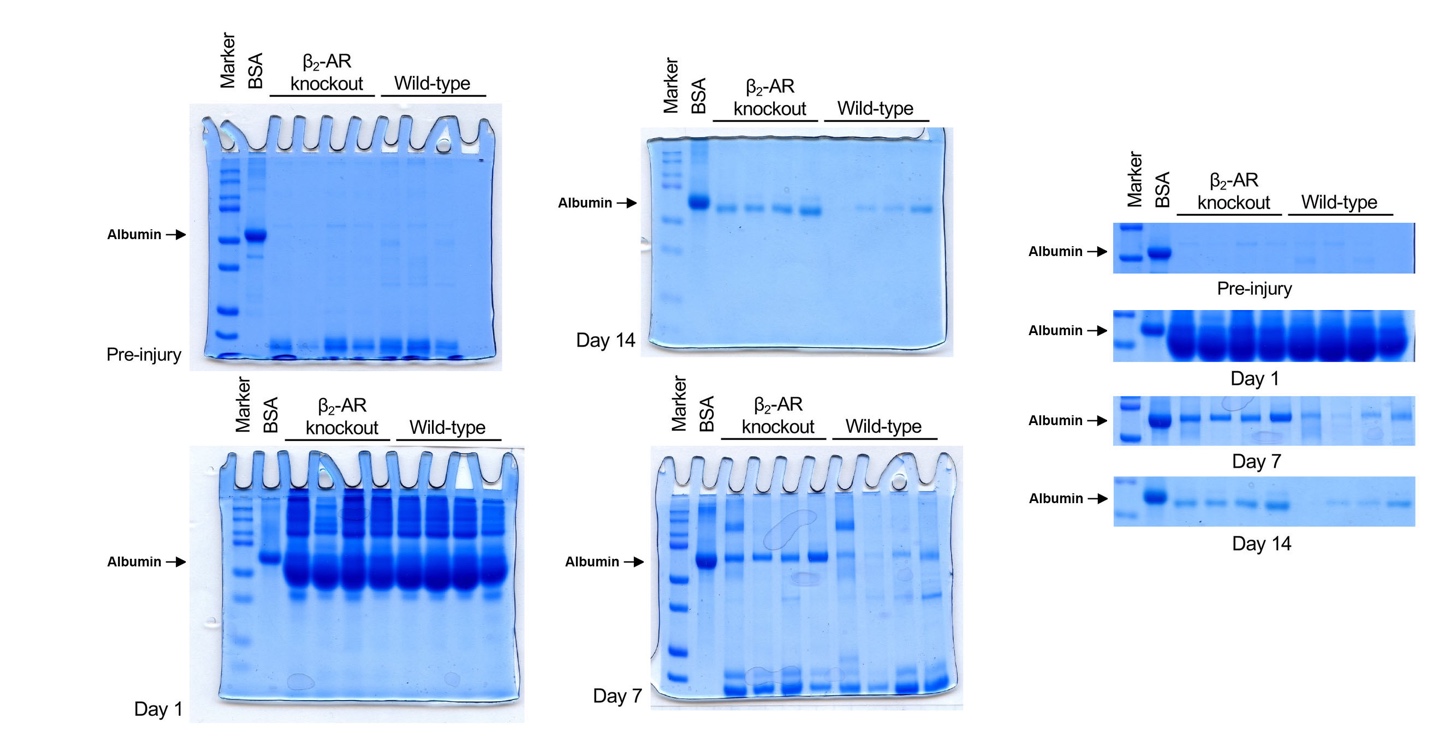


**Supplemental Figure 4: Whole image of Coomassie blue stained gels.** The whole gel images correspond to the data presented in Figure 3B.

**
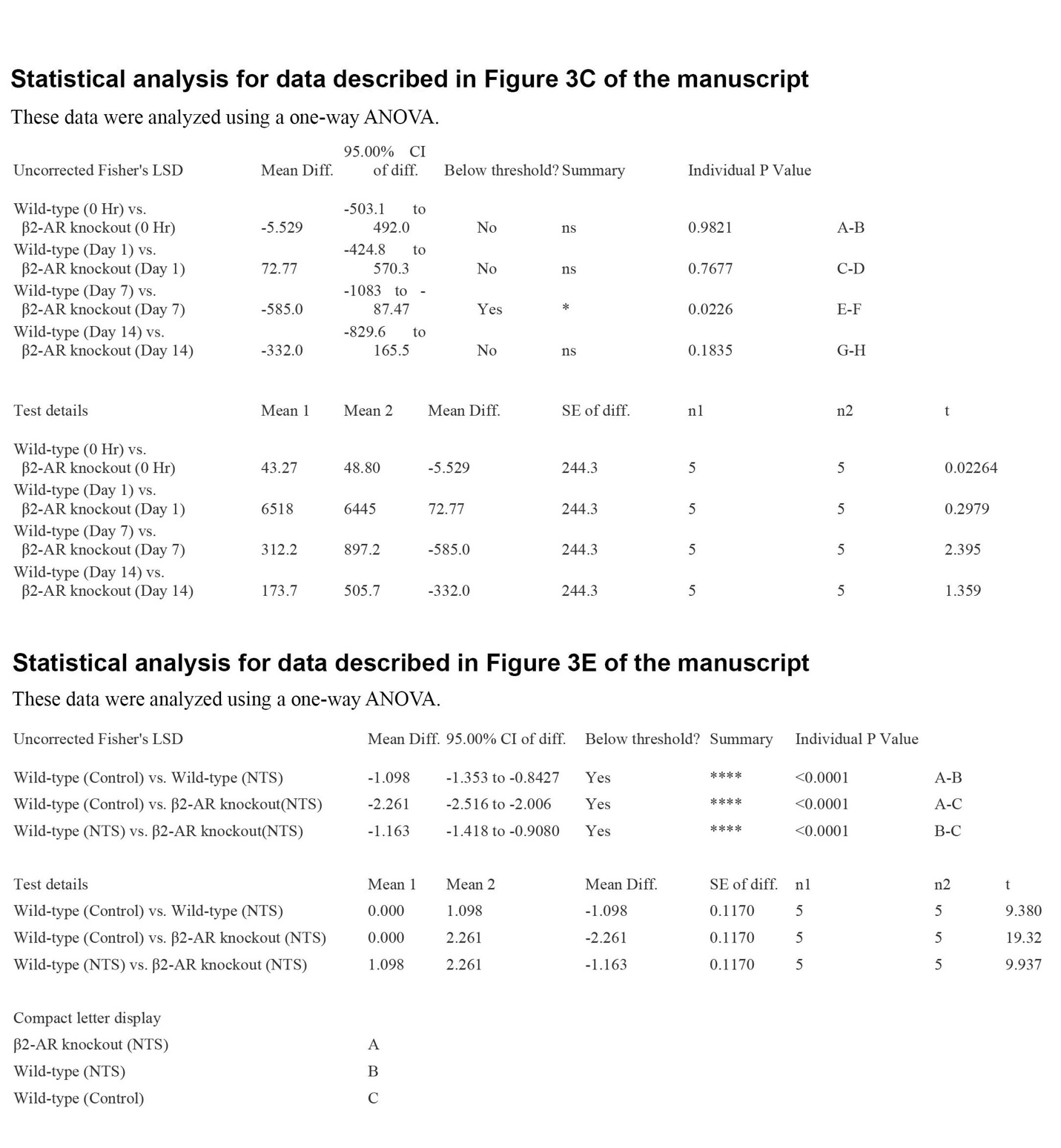
**

**Supplemental Figure 5: Statistical analyses for Figures 3C and 3E.**

**
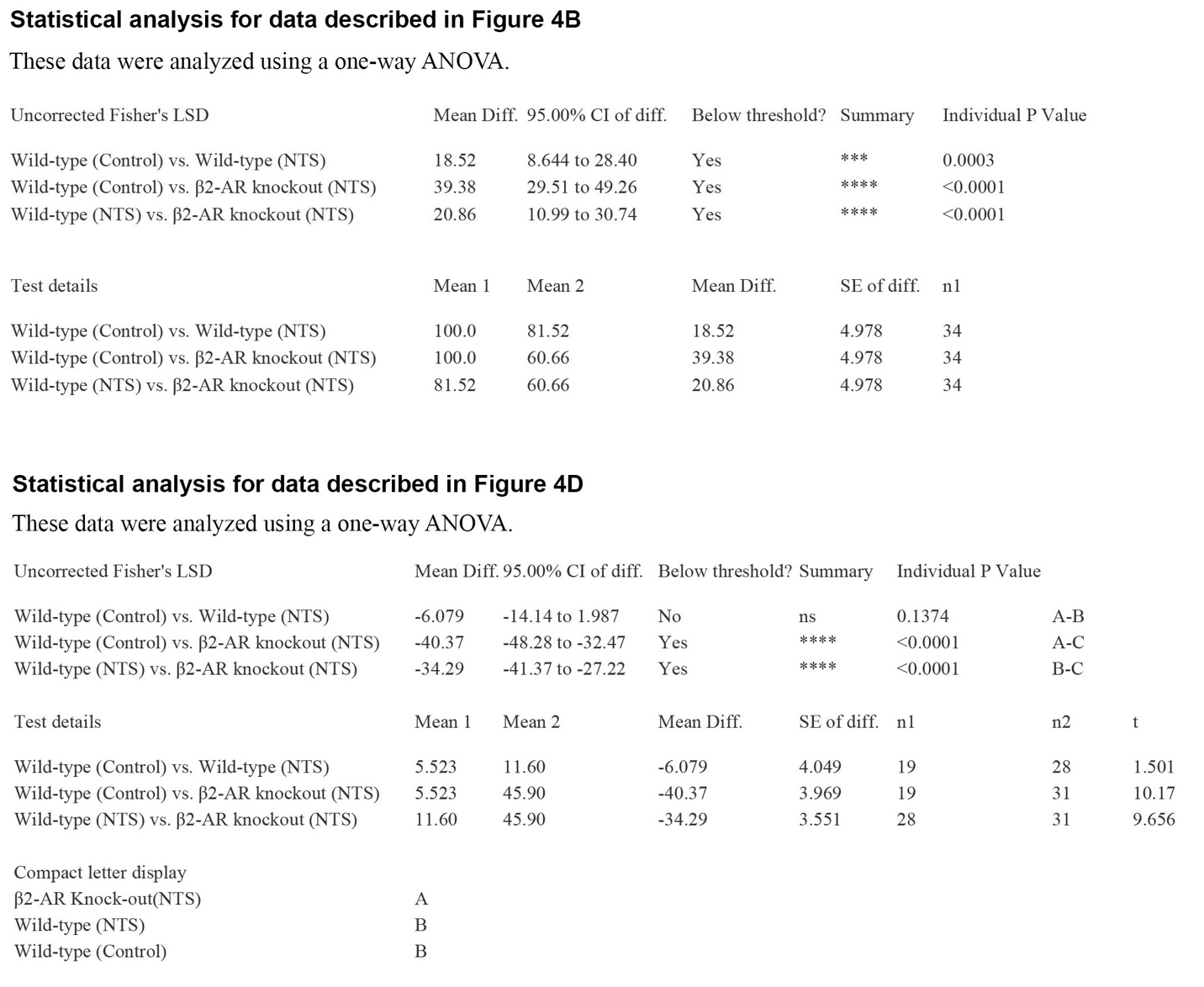
**

**Supplemental Figure 6: Statistical analyses for Figures 4B and 4D.**
